# Impact of tissue properties on time-dependent alterations in apparent diffusion coefficient: a phantom study using oscillating-gradient spin-echo and pulsed-gradient spin-echo sequences

**DOI:** 10.1007/s11604-022-01281-2

**Published:** 2022-05-07

**Authors:** Kazushige Ichikawa, Toshiaki Taoka, Masanori Ozaki, Mayuko Sakai, Hiroshi Yamaguchi, Shinji Naganawa

**Affiliations:** 1 Department of Radiological Technology, Nagoya University Hospital, 65 Tsurumai-cho, Showa-ku, Nagoya, Aichi 466-8560 Japan; 2 Department of Innovative Biomedical Visualization (iBMV), Nagoya University Graduate School of Medicine, 65 Tsurumai-cho, Showa-ku, Nagoya, Aichi 466-8560 Japan; 3 Department of Radiology, Nagoya University Graduate School of Medicine, 65 Tsurumai-cho, Showa-ku, Nagoya, Aichi 466-8560 Japan; 4 Canon Medical Systems Corporation, Otawara, Tochigi 324-8550 Japan; 5 Nagoya University Radioisotope Research Center Medical Branch, 65 Tsurumai-cho, Showa-ku, Nagoya, Aichi 466-8560 Japan

**Keywords:** ADC, Spatial restriction, Diffusion time, Oscillating-gradient spin-echo, Phantom

## Abstract

**Purpose:**

The purpose of this study was to investigate whether the changes in apparent diffusion coefficients (ADCs) due to differences in diffusion time reflect tissue properties in actual measurements of phantoms.

**Materials and methods:**

Various n-alkane phantoms and sucrose/collagen phantoms with various collagen densities were set up with and without polyvinyl alcohol (PVA) foam with an average pore diameter of 300 μm. Thus, n-alkanes or sucrose/collagen represented substrate viscosity and the presence of PVA foam represented tissue structure with septum. Diffusion-weighted images with various diffusion times (7.71–60 ms) were acquired using pulsed-gradient spin-echo (PGSE) and oscillating-gradient spin-echo (OGSE) sequences. The ADCs of the phantoms with and without PVA foam were calculated.

**Results:**

The ADCs of some of the phantoms without PVA decreased with diffusion times decreased. In the n-alkane phantoms, only C_8_H_18_ showed significantly different ADCs depending on the use of PVA foam in the OGSE sequence. On the other hand, sucrose/collagen phantoms showed significant differences according to diffusion time. The ADCs of the phantoms decreased as the molecular size of the n-alkanes or collagen density of the sucrose/collagen phantom increased. Compared to phantoms without PVA foam, the ADC of the phantoms with PVA foam decreased as the diffusion time increased.

**Conclusion:**

Changes in ADCs due to differences in diffusion time reflect tissue properties in actual measurements of phantoms. These changes in ADCs can be used for tissue characterization in vivo.

## Introduction

Diffusion-weighted imaging (DWI) is now an established non-invasive technique for investigating brain lesions such as acute ischemic stroke [1], encephalopathy [2], multiple sclerosis [3], and tumors [4]. Although many DWI methods have been developed, most DWI performed in clinical practice uses the pulsed-gradient spin-echo (PGSE) sequence that was originally introduced by Stejskal and Tanner [5]. The apparent diffusion coefficient (ADC) obtained using the PGSE sequence can probe the water displacement. ADCs are potentially influenced by various tissue characteristics, including the sizes of cells and viscosities of substrates [6], and pathology that alters the tissue structure, such as ischemia [7]. The diffusion time is another factor that alters the ADC. However, the PGSE sequence often uses relatively long diffusion times due to the limited capacity to shorten the diffusion time owing to hardware limitations; therefore, a short diffusion time cannot be applied using PGSE sequences. To overcome this problem, the oscillating-gradient spin-echo (OGSE) sequence has been developed to probe much shorter diffusion times using rapidly oscillating diffusion gradients [8]. As the diffusion time increases, water molecules interact with more barriers, and the observed ADC decreases asymptotically [9]. The OGSE sequence is reported to be used to estimate microstructures [7, 10] and cancer grades [11, 12] from changes in ADCs with different diffusion times. The effectiveness of ADCs using the OGSE sequence has been reported in both brain tumors [13] and breast cancers [14].

Tofts et al. developed an isotropic diffusion phantom [15]. They suggested that random errors in the diffusion coefficient measurements using n-alkanes (C_n_H_2n+2_, where n is the number of carbon atoms) were small. N-alkanes have linear structures consisting of a chain of methylene groups with a methyl (CH_3_) terminus at each end. The viscosity increases with the carbon number of n-alkanes [16]. Furthermore, Maekawa et al. reported that the n-alkane phantom is a suitable isotropic diffusion phantom for the evaluation of ADCs obtained by the OGSE sequence [17]. In their report, since there was no spatial restriction for molecule movement during the preset diffusion time, the ADCs obtained by the PGSE and OGSE sequences did not depend on the diffusion time [17]. However, when spatial restriction is imposed on n-alkanes, the ADCs obtained by the PGSE and OGSE sequences are expected to decrease as the diffusion time increases. Baron et al. [18] reported that the changes in ADCs due to differences in diffusion time could reflect tissue structure using a simulation. However, to the best of our knowledge, there are no reports on actual measurements of ADCs using phantoms.

The purpose of this study was to investigate whether the changes in ADC due to the difference in diffusion time reflect the tissue properties in measurements of phantoms. We utilized various n-alkane phantoms and sucrose/collagen phantoms with various collagen densities to represent different substrate viscosities in tissue. In these n-alkanes and sucrose/collage phantoms, we placed polyvinyl alcohol (PVA) foam with an average pore diameter of 300 μm to represent the septum structure in the tissue. The ADCs of the phantoms were measured using a PGSE sequence and an OGSE sequence with diffusion times ranging from 7.71 to 60 ms.

## Materials and methods

### Phantom preparation

#### N-alkane phantoms

**Fig. 1:**
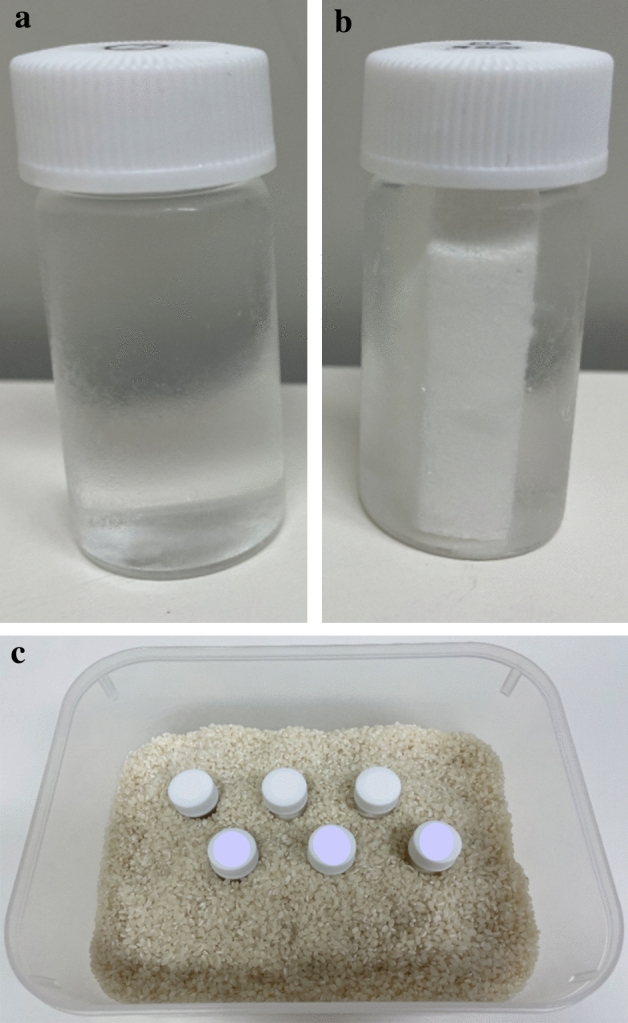
N-alkane phantoms. **a** A bottle containing only one n-alkane solution. **b** A bottle containing one of the n-alkane solutions and polyvinyl alcohol sponge. **c** To improve the local magnetic field uniformity, each of the phantoms was surrounded by rice

We used nine n-alkanes (Kanto Chemical Co., Tokyo, Japan), including n-octane (C_8_H_18_), n-nonane (C_9_H_20_), n-decane (C_10_H_22_), n-undecane (C_11_H_24_), n-dodecane (C_12_H_26_), n-tridecane (C_13_H_28_), n-tetradecane (C_14_H_30_), n-pentadecane (C_15_H_32_), and n-hexadecane (C_16_H_34_). Each n-alkane was put in a 13.5-mL borosilicate glass bottle without PVA foam (e.g., C_8_H_18_ PVA-free; Fig. 1a) and a bottle with PVA foam (Fuji Chemical Industries Ltd., Osaka, Japan) with an average pore diameter of 300 μm (e.g., C_8_H_18_ PVA-300; Fig. 1b). We made one each of these phantoms. The molecular size of the n-alkanes represented substrate viscosity, and the placement of PVA represented the viscosity of the tissue substance and the tissue structure.

#### Sucrose/collagen phantoms

Because the *T*_2_ values of n-alkanes are unrealistically long for human brain white matter [15], we made sucrose/collagen phantoms with *T*_2_ values close to that of normal white matter [19, 20]. By changing the mixing ratio of purified water (KOGA Chemical Mfg Co., Ltd., Saitama, Japan), sucrose (Hayashi Pure Chemical Ind., Ltd., Osaka, Japan), and collagen peptide (healthy company, Hiroshima, Japan), we made six solutions with different ADCs and *T*_2_ values close to that of normal white matter. The mixing ratios of the three agents and the *T*_2_ values are shown in Table 1. Purified water and each of the six solutions (#1–#6 shown in Table 1) were put in 13.5 mL borosilicate glass bottles in the same fashion as the n-alkane phantoms (e.g., #1 PVA-free, #1 PVA-300, etc.). PVA foam with an average pore diameter of 80 μm was used for purified-water phantoms in addition to 300-μm foam; thus, there were three types of purified-water phantoms (PVA-free, PVA-80, and PVA-300). We made one each of these phantoms. The collagen density of the sucrose/collagen phantoms represented substrate viscosity, and the PVA represented the septal structure of the tissue.

**Table 1:** The mixing ratio of purified water, sucrose, and collagen peptide, and T_2_ values

Phantoms	Purified water	#1	#2	#3	#4	#5	#6
Purified water	100	80	72	68	64	60	56
Sucrose	0	10	8	7	6	5	4
Collagen peptide	0	10	20	25	30	35	40
T_2_ values	2364	71	76	79	78	73	66

Mixing ratios are wt% of total

#### Preparation for acquisition

The PVA foam was placed in a bottle. The phantoms were deaerated with an oil-sealed rotary vacuum pump (GLD-051, ULVAC KIKO Inc., Yokohama, Japan) and a Vacuum Polyca Desiccator (240 Type-G2, AS ONE Corp., Osaka, Japan) to avoid susceptibility artifacts. To improve the uniformity of the local magnetic field, each of the phantoms was surrounded by rice (Fig. 1c). Phantoms were placed in the MRI scanning room in which the room temperature was controlled at 22 °C for more than 12 h to stabilize the temperature. The phantoms were placed close to the isocenter for approximately 15 min before the scan to eliminate the effects of flow.

### MRI acquisition and analysis

All MRI experiments were performed using a 3 T clinical scanner (Vantage Centurian, Canon Medical Systems, Tochigi, Japan) with two 16-channel Flex SPEEDER coils. The temperature of the phantoms in the magnet bore (with no temperature control other than room air conditioning) was 22 °C, as measured using a thermometer.

The common parameters for both the PGSE sequence and the trapezoid sine OGSE prototype sequence were as follows: repetition time (TR), 6000 ms; echo time (TE), 143 ms; echo spacing, 0.7 ms; band width, 1563 Hz/Px; field of view (FOV), 60 × 180 mm^2^; matrix size, 80 × 112; 3 slices with distance factor of 300%; slice thickness, 5 mm; number of averages, 6; acceleration factor, 1; acquisition time, 3.5 min; motion-probing gradient, monopolar type; *b* value, 0; 700 s/mm^2^; 3-axis mixed. We followed the same *b* values as previous study [17]. The specific parameters for the PGSE sequence were as follows: diffusion time (*T*_diff_) = 20, 40, and 60 ms. *T*_diff_ was defined as “large delta–small delta/3” (large delta = duration between the onset of the first and second pulses, small delta = pulse duration). The specific parameters for the OGSE sequence were as follows: oscillation frequency, 40, 30, and 21 Hz; number of oscillation cycles, 1, 1, and 1; and *T*_diff_, 7.71, 10.52, and 15.31 ms, respectively. *T*_diff_ was defined using the equations described in the Appendix. The phantoms were scanned five times in rows with identical acquisition parameters.

In addition, the *T*_2_ values of each sucrose/collagen phantom were measured using the following parameters: TR, 3000 ms; TE, 20, 40, 60, 80, 100, 120, 140, 160, 180, and 200 ms; band width, 195 Hz/Px; FOV, 250 × 250 mm^2^; matrix size, 128 × 128; slice thickness, 5 mm; number of averages, 1; acquisition time, 4 min.

In the measurement of ADCs, circular regions of interest (ROIs) were drawn manually on the console twice four months apart by a radiological technologist, and the mean and standard deviation of the five measurements within the ROIs were calculated. Each ROI consisted of 36 pixels.

### Statistical analysis

The reproducibility of ADC measurements and statistical significance whether ADC values depend on diffusion times were calculated using Pearson’s product-moment correlation function.

We investigated whether the mean ADCs of PVA-free phantoms were statistically higher than those of PVA-300 phantoms at each diffusion time using Welch’s *t *test. Statistical significance was set at *p* < 0.05. All statistical analyses were performed using R version 4.1.0 software (R Foundation for Statistical Computing, Vienna, Austria.).

## Results

**Fig. 2:**
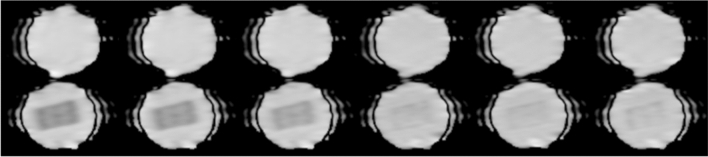
The images of purified-water phantoms. Upper row, PVA-free; lower row, PVA-300. From left column to right column of the images, diffusion time = 60, 40, 20, 15.31, 10.52, and 7.71 ms

We were able to obtain all the images with identical acquisition parameters. As an example, the images of purified-water phantoms are shown in Fig. 2.

### N-alkane phantoms

The reproducibility of ADC measurements for the n-alkane phantoms was statistically significant (*r* = 0.9996, *p* < 0.001).

**Fig. 3:**
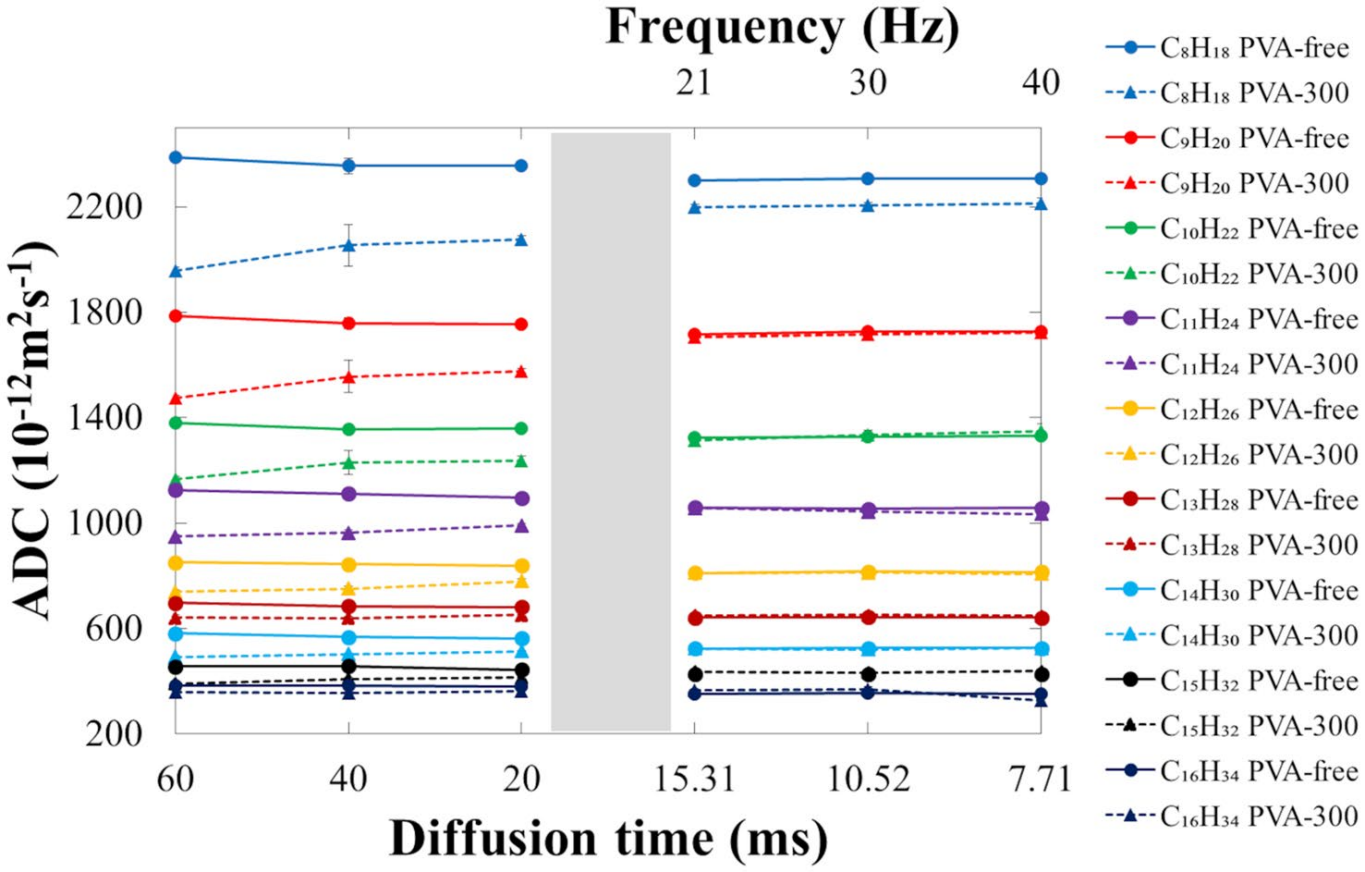
The mean apparent diffusion coefficients of the n-alkanes phantoms with different diffusion times. In all the n-alkane phantoms with PVA free, the ADC values decreased as the diffusion time decreased (*p* < 0.05). In all the n-alkane phantoms except C_16_H_34_ with PVA-300, the ADC values increased as the diffusion time decreased (*p* < 0.05). In the PGSE sequence, the mean ADCs of the PVA-free phantoms were significantly higher (*p* < 0.05) than those with PVA-300 at each diffusion time. In the OGSE sequence, none of the mean ADCs of the n-alkane phantoms, except C_8_H_18_, were not significantly different. There were few differences in the mean ADCs between PVA-free and PVA-300 conditions

The mean ADCs of the n-alkane phantoms for each diffusion time are listed in Table 2. In all the n-alkane phantoms with PVA free, the ADC values decreased as the diffusion time decreased (*p* < 0.05) (Fig. 3). In all the n-alkane phantoms except C_16_H_34_ with PVA-300, the ADC values increased as the diffusion time decreased (*p* < 0.05). In the PGSE sequence, the mean ADCs of the PVA-free n-alkane phantoms were significantly higher (*p* < 0.05) than those with PVA-300 at each diffusion time, including 20, 40, and 60 ms. However, in the OGSE sequence with diffusion times of 7.71, 10.52, and 15.31 ms, none of the mean ADCs of the n-alkane phantoms, except for C_8_H_18_, were significantly different between PVA-free and PVA-300 conditions. Compared with the PVA-free n-alkane phantoms, the mean ADCs of those with PVA-300 decreased as the diffusion time increased.

**Table 2:** Apparent diffusion coefficients of each n-alkane phantoms for each diffusion time

*T*_diff_ (ms)	Apparent diffusion coefficient (10^−12^m^2^s^−1^)									
	C_8_H_18_		C_9_H_20_		C_10_H_22_		C_11_H_24_		C_12_H_26_	
	PVA-free	PVA-300	PVA-free	PVA-300	PVA-free	PVA-300	PVA-free	PVA-300	PVA-free	PVA-300
60	2387 ± 17	1956 ± 15	1786 ± 2	1476 ± 7	1378 ± 5	1165 ± 13	1125 ± 23	949 ± 7	851 ± 6	739 ± 7
	*		*		*		*		*	
40	2355 ± 30	2054 ± 79	1759 ± 20	1556 ± 61	1356 ± 17	1230 ± 45	1112 ± 19	963 ± 10	843 ± 5	751 ± 11
	*		*		*		*		*	
20	2355 ± 12	2076 ± 14	1754 ± 2	1577 ± 8	1358 ± 4	1238 ± 14	1096 ± 11	991 ± 7	839 ± 4	779 ± 8
	*		*		*		*		*	
15.31	2301 ± 3	2199 ± 8	1716 ± 2	1705 ± 8	1325 ± 1	1314 ± 6	1059 ± 2	1058 ± 4	811 ± 1	810 ± 3
	*		*		*		ns		ns	
10.52	2308 ± 4	2206 ± 8	1725 ± 2	1715 ± 5	1329 ± 1	1335 ± 15	1054 ± 1	1044 ± 3	816 ± 2	813 ± 3
	*		*		ns		*		ns	
7.71	2305 ± 4	2212 ± 21	1728 ± 2	1722 ± 10	1329 ± 1	1349 ± 29	1057 ± 1	1035 ± 5	815 ± 1	807 ± 4
	*		ns		ns		*		*	

*T*_diff_ (ms)	Apparent diffusion coefficient (10^−12^m^2^s^−1^)							
	C_13_H_28_		C_14_H_30_		C_15_H_32_		C_16_H_34_	
	PVA-free	PVA-300	PVA-free	PVA-300	PVA-free	PVA-300	PVA-free	PVA-300
60	697 ± 5	642 ± 9	582 ± 5	493 ± 5	456 ± 1	391 ± 3	383 ± 1	358 ± 12
	*		*		*		*	
40	685 ± 4	639 ± 7	568 ± 6	503 ± 3	456 ± 2	408 ± 4	384 ± 3	353 ± 11
	*		*		*		*	
20	680 ± 2	652 ± 6	562 ± 3	513 ± 2	444 ± 1	414 ± 4	379 ± 2	361 ± 13
	*		*		*		*	
15.31	640 ± 1	649 ± 4	523 ± 1	524 ± 1	425 ± 1	436 ± 4	351 ± 1	364 ± 2
	ns		ns		ns		ns	
10.52	643 ± 2	651 ± 2	525 ± 1	520 ± 3	427 ± 1	433 ± 2	353 ± 1	370 ± 10
	ns		*		ns		ns	
7.71	640 ± 1	648 ± 1	525 ± 1	525 ± 4	426 ± 1	438 ± 2	352 ± 1	327 ± 4
	ns		ns		ns		*	

Values are mean ± standard deviation within the region of interest

*T*_diff_ diffusion time, *PVA* polyvinyl alcohol, *ns* not significant

*p < 0.05

### Sucrose/collagen phantoms

The reproducibility of ADC measurements for the sucrose/collagen phantoms was statistically significant (*r* = 0.9994, *p* < 0.001).

**Fig. 4:**
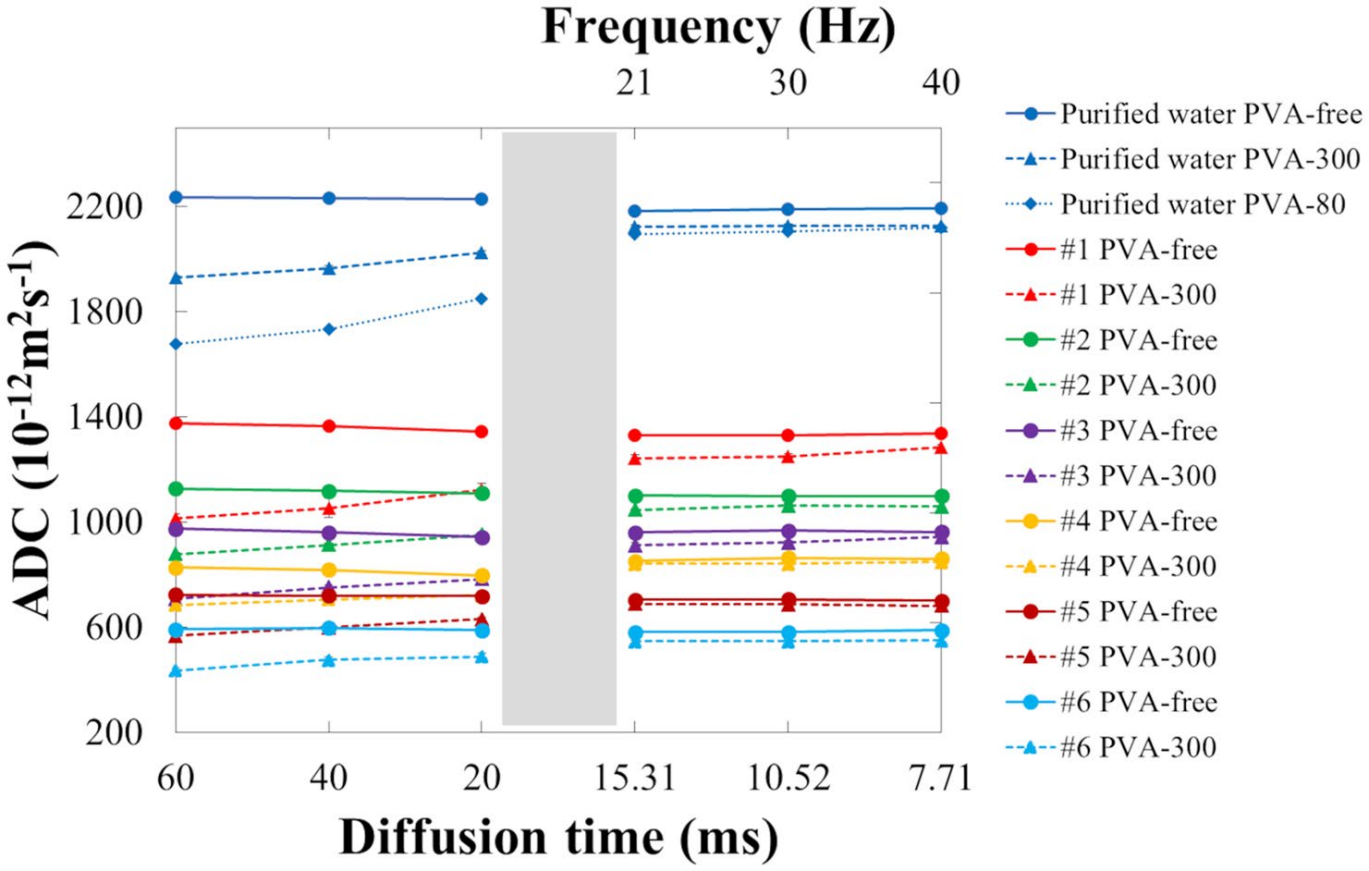
The mean apparent diffusion coefficients of sucrose/collagen phantoms with different diffusion times. In the purified-water phantom and some sucrose/collagen phantoms with PVA free (#1, #2), the ADC values decreased as the diffusion time decreased (*p* < 0.05). In all the sucrose/collagen phantoms with PVA-300, the ADC values increased as the diffusion time decreased (*p* < 0.05). For all diffusion times, the mean ADCs of the PVA-free phantoms were significantly higher (*p* < 0.05) than those with PVA-300

The mean ADCs of each of the sucrose/collagen phantoms for each diffusion time are shown in Table 3 and Fig. 4. In the purified-water phantom and some sucrose/collagen phantoms with PVA free (#1, #2), the ADC values decreased as the diffusion time decreased (*p* < 0.05) (Fig. 4). In all the sucrose/collagen phantoms with PVA-300, the ADC values increased as the diffusion time decreased (*p* < 0.05) (Fig. 4). In each of the diffusion times, the mean ADCs of the PVA-free sucrose/collagen phantoms were significantly higher (*p* < 0.05) than those of PVA-300. Compared with the PVA-free sucrose/collagen phantoms, the mean ADCs of those with PVA-300 decreased as the diffusion time increased.

**Table 3:** Apparent diffusion coefficients of each sucrose/collagen phantom for each diffusion time

*T*_diff_ (ms)	Apparent diffusion coefficient (10^−12^m^2^s^−1^)						
	Purified water			#1		#2	
	PVA-free	PVA-300	PVA-80	PVA-free	PVA-300	PVA-free	PVA-300
60	2238 ± 7	1930 ± 6	1678 ± 3	1376 ± 7	1017 ± 15	1128 ± 2	878 ± 5
	*			*		*	
40	2231 ± 7	1967 ± 7	1735 ± 2	1365 ± 7	1055 ± 36	1120 ± 2	914 ± 5
	*			*		*	
20	2228 ± 4	2026 ± 7	1851 ± 2	1345 ± 6	1124 ± 23	1110 ± 12	952 ± 6
	*			*		*	
15.31	2184 ± 3	2123 ± 8	2097 ± 1	1330 ± 5	1242 ± 15	1102 ± 8	1048 ± 3
	*			*		*	
10.52	2192 ± 2	2128 ± 6	2105 ± 3	1332 ± 4	1252 ± 10	1100 ± 5	1063 ± 7
	*			*		*	
7.71	2196 ± 1	2126 ± 3	2121 ± 4	1337 ± 3	1284 ± 11	1100 ± 3	1061 ± 4
	*			*		*	

*T*_diff_ (ms)	Apparent diffusion coefficient (10^−12^m^2^s^−1^)							
	#3		#4		#5		#6	
	PVA-free	PVA-300	PVA-free	PVA-300	PVA-free	PVA-300	PVA-free	PVA-300
60	976 ± 2	708 ± 12	828 ± 3	685 ± 9	724 ± 2	569 ± 10	593 ± 3	437 ± 7
	*		*		*		*	
40	961 ± 1	752 ± 10	818 ± 1	707 ± 8	721 ± 2	600 ± 4	597 ± 2	477 ± 10
	*		*		*		*	
20	944 ± 3	784 ± 12	798 ± 5	723 ± 10	719 ± 3	632 ± 8	589 ± 2	490 ± 11
	*		*		*		*	
15.31	960 ± 2	912 ± 13	852 ± 3	841 ± 8	704 ± 2	690 ± 4	582 ± 4	547 ± 4
	*		*		*		*	
10.52	968 ± 2	923 ± 6	864 ± 3	842 ± 12	707 ± 2	687 ± 6	583 ± 1	547 ± 8
	*		*		*		*	
7.71	963 ± 4	944 ± 6	862 ± 1	848 ± 5	701 ± 3	683 ± 7	589 ± 1	551 ± 19
	*		*		*		*	

Values are mean ± standard deviation within the region of interest

*T*_diff_ diffusion time, *PVA* polyvinyl alcohol

*p < 0.05

## Discussion

In the current study, we investigated whether changes in ADCs by diffusion time reflect tissue properties in actual measurements of phantoms. In all the n-alkane phantoms with PVA free, the ADC values decreased as the diffusion time decreased (*p* < 0.05). In the phantoms with PVA free, the ADC values should be constant independent of the diffusion times, however, our study was not constant on varying diffusion times. It has been reported that this is because an OGSE sequence essentially has motion compensation, while a conventional PGSE sequence does not [21]. It was thought that the effect of the vibration of the magnetic gradient coil was cancelled to some extent in an OGSE sequence, but not in a PGSE sequence, resulting in a higher ADC with diffusion time. We think this is the reason why our study did not show a constant independent of diffusion times. In all the n-alkane phantoms except C_16_H_34_ with PVA-300, the ADC values increased as the diffusion time decreased (*p* < 0.05). Because C_16_H_34_ was the lowest ADC phantom including the sucrose/collagen phantoms, we might not have successfully deaerated it. In the n-alkane phantoms with PVA-300, the mean ADCs obtained by the PGSE sequence decreased as the diffusion time increased, as expected. In contrast, the mean ADCs obtained using the OGSE sequence were almost constant. This was thought to be because the difference in diffusion time was smaller for the OGSE sequence (*T*_diff_ = 7.71–15.31 ms) than for the PGSE sequence (*T*_diff_ = 20–60 ms), and, therefore, the difference in diffusion distance was smaller. In the PGSE sequence acquisition for n-alkane phantoms, the mean ADCs of the PVA-free n-alkane phantoms were significantly higher (*p* < 0.05) than those with PVA-300 for all diffusion times. This was thought to be because the diffusion of molecules in the n-alkane phantoms with PVA-300 was spatially restricted and molecules interacted with more barriers. In the OGSE sequence acquisition for n-alkane phantoms, none of the mean ADCs of the n-alkane phantoms, except C_8_H_18_, showed significant differences between PVA-free and PVA-300 conditions. But, this difference remained unclear in this study, so further study was necessary to investigate. In the purified-water phantom and some sucrose/collagen phantoms with PVA free (#1, #2), the ADC values decreased as the diffusion time decreased (*p* < 0.05). Since a PGSE sequence does not have motion compensation, it was considered that the higher the ADC value, the more the phantom was affected by diffusion time. In each of the diffusion times, the mean ADCs of the PVA-free sucrose/collagen phantoms were significantly higher (*p* < 0.05) than those with PVA-300. In the n-alkane phantoms, only C_8_H_18_ showed significant difference in ADC values between PVA-free and PVA-300 in the OGSE sequence. In the sucrose/collagen phantoms, however, all phantoms showed significant difference in ADC values between PVA-free and PVA-300. Commercially available collagen peptides have broad molecular weight distributions ranging from 2 to 20 kDa [22]. This difference might reflect the fact that the sucrose/collagen phantoms were more non-uniform than the n-alkane phantoms. Most of the mean ADCs, and especially high ADCs, in the PVA-free phantoms were higher in the PGSE sequence than in the OGSE sequence for sucrose/collagen phantoms. As mentioned above, this seems to be due to the absence of compensation for the motion of water molecules in the PGSE sequence [21].

In the current study, we prepared sucrose/collagen phantoms with PVA with an average pore diameter of 300 μm. The characteristic large cystic cavitation observed in Creutzfeldt–Jakob disease (CJD) at stage VI is approximately 300 μm in diameter [23]. Thus, our phantoms with PVA-300 may simulate the tissue structure observed in CJD. A previous study used a simulation method to compare ADCs of “bead + swell” (where it was regarded as more spatially restricted than the “healthy” condition) and “healthy” conditions using PGSE and OGSE sequences [18]. The ADCs of the bead + swell condition compared to the healthy condition were reduced by 21% using the OGSE sequence (*T*_diff_ = 4 ms), in contrast to a 37% decrease using the PGSE sequence (*T*_diff_ = 40 ms). In our study, for example, in the #4 sucrose/collagen phantoms, the mean ADCs of the sucrose/collagen phantoms with PVA-300 compared to those without PVA were reduced by 2% using the OGSE sequence (*T*_diff_ = 7.71 ms), in contrast to a 14% decrease in the PGSE sequence (*T*_diff_ = 40 ms). Our results were consistent with those of Baron et al. [18], who used simulations in which the decrease was smaller at short diffusion times.

Our study had several limitations. First, the average pore diameter of our PVA phantom was 300 µm, and we could only apply the 80 μm PVA in purified water. Of note, even the 80 μm pore diameter was larger than those in previous simulation studies [24]. However, deaeration of the PVA sponge with viscous liquid was very difficult for 80 μm PVA; thus, only the purified-water phantom could be used 80 μm PVA. It may be desirable to use a phantom with a smaller pore diameter in the future. Second, there may have been an effect of vibration during scanning. Because the phantoms were on the table of MR scanner, the ADCs were thought to be affected by the vibrations to some extent. Third, when water molecules collided with the PVA foam within the diffusion time, the possibility of water molecules passing through the PVA foam could not be excluded. Fourth, the sponges used in this study were standardized to have a pore diameter of 300 microns. However, not all of the pores were 300 microns, but rather the average of a mixture of pores of various sizes. The mixture of large and small pores may have prevented us from obtaining the observed values as predicted by theory. And, therefore, the ADCs obtained in this study should be considered with some caution.

In conclusion, we have shown that changes in ADCs due to differences in diffusion time reflect tissue properties in measurements of phantoms. The mean ADCs of the phantoms decreased as the molecular size of the n-alkanes or the collagen density of the sucrose/collagen phantom increased. Compared to PVA-free phantoms, the mean ADCs of the phantoms with PVA-300 representing tissue with septum structure showed a decrease as diffusion time increased. These differences in ADCs due to changes in diffusion time can be used for tissue characterization in vivo.

## Definition of diffusion time for the trapezoid sine OGSE sequence

The b value for the trapezoid sine oscillatory-gradient spin-echo (OGSE) sequence is defined as follows [8]:

1 $$b = \frac{{NG^{2} \gamma^{2} \delta }}{{30f^{2} }}\left( {5 - 15t_{r} f - 5t_{r}^{2} f^{2} + 32t_{r}^{3} f^{3} } \right),$$

where *N* is the number of periods (even number), *G* is the gradient amplitude, *γ* is, *δ* is the pulse duration, *f* is the oscillating frequency of the motion-probing gradient (MPG), and *t*_*r*_ is the ramp time of the MPG. The *q* value is defined by the following equation:

2 $$q = \left( {2\pi^{ - 1} } \right)\gamma G\left( {\frac{1}{2f} - \frac{{t_{r} }}{2}} \right).$$

Therefore, we can re-define the b value of the trapezoid sine OGSE using Eq. 2 as following,

3 $$b = \left( {2\pi } \right)^{2} Nq^{2} T_{{{\text{diff}}}} ,$$

where *T*_diff_ is the diffusion time; thus, the diffusion time of the trapezoid sine OGSE is calculated using Eqs. 1, 2, and 3.
